# Coffee Consumption and the Risk of Obesity in Korean Women

**DOI:** 10.3390/nu9121340

**Published:** 2017-12-08

**Authors:** Jeonghee Lee, Hye Young Kim, Jeongseon Kim

**Affiliations:** 1 Department of Cancer Biomedical Science, Graduate School of Cancer Science and Policy, National Cancer Center, Goyang-si 10408, Korea; jeonghee@ncc.re.kr (J.L.); hypkim@yongin.ac.kr (H.Y.K.); 2 Department of Foods and Nutrition, Yongin University, Yongin 17092, Korea

**Keywords:** coffee, obesity, body mass index, waist circumference

## Abstract

Instant coffee mixes that contain sugar and non-dairy creamer account for 80–90% of the total coffee market in Korea. The objective of this study was to investigate the relationship between coffee consumption and obesity in Korean women. We included 5995 women who participated in a health screening examination at the Korean National Cancer Center between 2007 and 2016. Daily coffee consumption and the use of sugar and creamer in coffee was evaluated using a 106-item food frequency questionnaire. Obesity was assessed by body mass index (BMI), and abdominal obesity was assessed by waist circumference (WC). A multiple logistic regression model was used to calculate the odds ratio (OR) of obesity according to coffee consumption. After multivariate adjustment, high coffee consumption was positively associated with obesity as measured by BMI (≥3 cups vs. no drinks, OR = 2.52; 95% confidence interval (CI) = 1.91–3.34; *p* for the trend < 0.001) and abdominal obesity as measured by WC (≥3 cups vs. no drinks, OR = 2.11; 95% CI = 1.59–2.79; *p* for the trend < 0.001). The positive association between daily coffee consumption and obesity prevalence was not altered by menopause. The amount of coffee with additives consumed per day by Korean women was positively correlated with the prevalence of obesity, but causation cannot be determined due to the cross-sectional nature of the study design. The mechanism underlying the observed relationship is yet to be elucidated.

## 1. Introduction

Obesity is a major global public health problem. The World Health Organization (WHO) describes obesity as a global epidemic due to the rapid increase in the number of obese people [1]. In 2014, approximately 53% of adults in the world were overweight or obese [2]. Risks of hypertension, cardiovascular disease, type 2 diabetes mellitus and some types of cancer steadily increase with increasing body mass index (BMI) [3,4,5]. Mortality rates also increase with higher degrees of overweight [6]. Obesity is influenced by many dietary factors, including an increase in beverage consumption [6,7]. Increased sugar-sweetened beverage or fruit juice intake has been found to be associated with increased weight gain in women [7,8].

Coffee is one of the most popular beverages in the world. As eating habits have become westernized and lifestyles have changed, the culture of drinking coffee has become common in Korea [9]. Steady increases in the quantity of coffee imports and the consumption of coffee have been reported [10]. The average frequency of coffee consumption by Korean adults have increased from 9 times per week in 2008 to 12 times per week in 2015 [11]. 

The influence of coffee on human health and disease has long been a topic of interest [12,13]. Coffee contains several bioactive chemicals, such as caffeine, chlorogenic acid, and diterpenes, which have various effects on the human body [14]. Caffeine increases heat production and lipid peroxidation to increase weight loss, chlorogenic acid positively affects glucose metabolism, and diterpenes exert anti-inflammatory effects [15,16]. 

For many years, epidemiological studies investigating the association between coffee drinking and obesity based on BMI and waist circumference (WC) yielded inconsistent results. Coffee consumption was reported to be effective in preventing obesity by decreasing body weight and BMI in some studies [17,18,19,20,21], but other studies reported an increase in BMI and WC as coffee consumption increased [22,23]. Several other studies did not observe an association between coffee intake and obesity risk [24,25,26,27]. 

Because styles of drinking coffee vary by country and culture [12,28], research results from other countries are not directly applicable to people in Korea. Traditionally, Koreans favor drinking coffee with sugar and creamer added. Instant coffee mixes that contain sugar and non-dairy creamer account for 80–90% of the total coffee market in Korea [10]. Few studies have investigated the relationship between consumption of coffee with additives and obesity. Therefore, in this study, we aimed to evaluate the BMI and WC of Korean women based on their daily consumption of coffee with additives and to investigate the relationship between the consumption of coffee with additives and obesity risk. A subgroup analysis stratified by menopausal status was also performed to examine the possibility that menopause serves as a moderating variable.

## 2. Materials and Methods

### 2.1. Study Population

**Figure 1:**
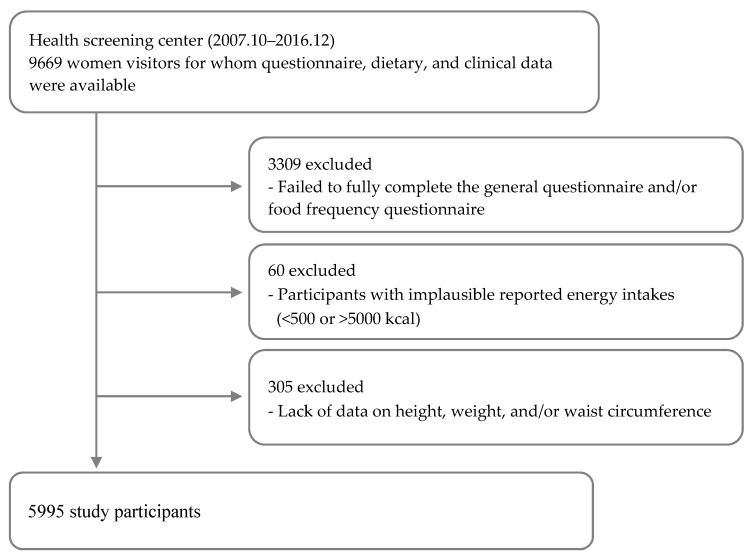
Flow chart of study selection process.

A total of 9669 female participants were recruited from a health screening examination at the National Cancer Center (NCC) in South Korea between October 2007 and December 2016. Three thousand three hundred nine participants who failed to complete the general questionnaire and food frequency questionnaire (FFQ) as well as participants with daily energy intakes of <500 kcal or >5000 kcal (*n* = 60) were excluded from the analysis. Information about height, weight, and WC were missing for 305 participants, who were also excluded. As a result, 5995 female participants ranging in age from 30 to 70 years old were included in the final analysis (Figure 1). The study protocol was approved by the Institutional Review Board (IRB) of the NCC (IRB No. NCCNCS-07-077). All participants provided written informed consent and ethical approval for publication prior to participation. The study protocols were performed according to the guidelines and regulations of the IRB of the NCC.

### 2.2. Data Collection

Each participant was asked to complete a self-administered questionnaire about her sociodemographic characteristics (e.g., age, education, and occupation), cigarette smoking habits, alcohol consumption, regular exercise habits, menstrual history, and history of hormone therapy. Usual dietary intake was assessed with the validated 106-item FFQ [29] over the past year, which included coffee consumption and the use of sugar and creamers in coffee. All subjects were interviewed about their average frequencies of intake and portion sizes of specific foods during the previous year. Consumption frequencies were divided into 9 categories: seldom or never, once a month, 2–3 times a month, 1–2 times a week, 3–4 times a week, 5–6 times a week, once a day, twice a day and 3 times a day. Portion sizes were classified into 3 categories: small, medium, and large. The average coffee consumption was calculated according to the standard portion size used in the study and then converted to daily intake. Coffee, coffee sugar, and coffee cream intake were computed by multiplying the frequency of consumption of each item by portion size. For simplicity, average coffee consumption was divided into none, <1 cup a day, 1 to <2 cups a day, 2 to <3 cups a day, and ≥3 cups a day. Daily nutrient intakes and total calorie intake were determined using CAN-Pro 4.0 (Computer Aided Nutritional Analysis Program, The Korean Nutrition Society, Seoul, Korea).

At the time of the screen, body weight was measured to the nearest 0.1 kg when subjects were wearing light clothes. Height was measured to the nearest 0.1 cm when subjects were standing without shoes. The height and weight of each subject were determined using the height & weight scale DS-103 (Dong Sahn Jenix, Seoul, Korea). BMI was calculated as weight in kilograms divided by the square of height in meters (kg/m^2^). WC was measured to the nearest 0.1 cm using a measuring tape above the umbilicus at minimal respiration. In the present study, a BMI greater than 25 kg/m^2^ and a waist circumference greater than 80 cm indicated obesity and abdominal obesity, respectively, according to the WHO Asian-Pacific guidelines [30].

### 2.3. Statistical Analysis

Categorical variables are presented as frequencies and percentages, and continuous variables are shown as the means and standard deviations (SDs). The *p* value for trends was calculated using Mantel–Haenszel chi-square tests for the categorical variables and generalized linear models (GLM) for continuous variables. Differences in the means for BMI and WC among subgroups with different levels of coffee consumption were statistically tested with GLM. GLM was also used to estimate the adjusted means and proportions among subgroups with different levels of coffee consumption after adjusting for covariates. To assess the association between coffee consumption and the prevalence of obesity, multiple logistic regression models were used to calculate odds ratios (ORs) and 95% confidence intervals (CIs). Model 1 was unadjusted. Model 2 was adjusted for age (continuous), education level (less than middle school, high school, or college or more), occupation (managers and professionals, office workers, laborers, or not in the labor force), alcohol consumption (non-drinker, ex-drinker, or current drinker), smoking status (non-smoker, ex-smoker, or current smoker), regular exercise (no or yes), and total calorie intake (continuous). Model 3 was adjusted for the covariates in Model 2 in addition to the use of sugar and creamer additives. We conducted subgroup analyses of coffee consumption and obesity risk stratified by menopausal status and implemented an additional adjustment for postmenopausal hormone use in the analysis of postmenopausal women. Linear trends across the coffee consumption categories were tested by assigning the median value of the category (0, 0.25, 1, 2, or 3 cups/day) to each participant and modeling this value as a continuous variable. SAS 9.4 software (SAS Institute, Inc., Cary, NC, USA) was used to perform the calculations, and a 2-sided *p* value less than 0.05 was considered statistically significant.

## 3. Results

The general characteristics of the study participants stratified by coffee consumption category are shown in Table 1. Participants with the highest coffee consumption (3 or more cups/day) tended to be younger (mean 49.7 years), have more education, have lower unemployment rates, be current drinkers, be current smokers and exercise less regularly. They also tended to have a higher total energy intake and used more coffee additives, such as sugar and creamer.

The anthropometric measurements of the study participants stratified by coffee consumption category are shown in Table 2. Significantly positive trends across coffee consumption frequency were observed for height, weight, BMI, and WC. The results were similar for Model 2, which was adjusted for age, education level, occupation, alcohol intake, smoking status, regular exercise and total energy intake. However, height did not significantly differ among the coffee consumption groups. Model 3 was further adjusted for the use of sugar and creamer additives, and significantly positive trends in weight, BMI, and WC with increasing frequency of coffee consumption were observed.

Table 3 presents the OR for the prevalence of obesity as defined by BMI (≥25) in relation to coffee consumption. In the multivariate logistic regression model, high coffee consumption was positively associated with obesity (≥3 cups vs. no drinks, OR = 2.52; 95% CI = 1.91–3.34; *p* for the trend < 0.001). After stratifying participants by menopausal status, a positive association between coffee consumption and BMI remained for both premenopausal (OR = 2.28, 95% CI = 1.36–3.82; *p* for the trend = 0.006) and postmenopausal (OR = 2.52, 95% CI = 1.79–3.54; *p* for the trend < 0.001) women.

Table 4 shows the OR for the prevalence of abdominal obesity as defined by WC in relation to coffee consumption. In the multivariate logistic regression model, high coffee consumption was positively associated with obesity (≥3 cups vs. no drinks, OR = 2.11; 95% CI = 1.59–2.79; *p* for the trend < 0.001). After stratifying participants by menopausal status, a positive association with abdominal obesity remained for both premenopausal (OR = 2.82, 95% CI = 1.55–5.12; *p* for the trend = 0.010) and postmenopausal (OR = 1.90, 95% CI = 1.36–2.67; *p* for the trend = 0.001) women.

## 4. Discussion

This study investigated the relationship between coffee consumption and obesity using BMI and WC in Korean women aged 30–70 years. Women who consumed coffee more than 3 times per day exhibited significantly greater BMI and WC values than did women who were not coffee drinkers after adjusting for age, education level, occupation, alcohol intake, smoking status, regular exercise, total energy intake, and the use of sugar and creamer additives. After stratifying participants by menopausal status, a positive association between coffee consumption and obesity remained for both premenopausal and postmenopausal women.

In several previous studies, coffee consumption was not related to obesity indices, such as BMI or WC [24,25,26,27]. In a cross-sectional study of 3823 National Health and Nutrition Examination Survey participants in the United States, coffee consumption was not associated with BMI or WC in either men or women. However, the BMI was higher among people who used artificial sweeteners in their coffee [25]. In a longitudinal study of Dutch people, coffee intake was not related to BMI and WC [26].

However, a cross-sectional study of 8821 people in Poland reported that the prevalence of obesity was lower in participants who drank more than three cups of coffee a day than in participants who drank less than one cup of coffee a day [18]. A cross-sectional study of 1902 Japanese men and women over age 40 also showed an inverse relationship between coffee consumption and WC [17]. 

Furthermore, in a prospective study of 14,629 Finnish men and women, BMI for both men and women increased with increasing coffee intake [22]. According to the results from a prospective study of Swedish women, the group who consumed more than 6 cups of coffee daily tended to have a higher BMI than the group who consumed less than 2 cups daily [23]. In a cross-sectional study of Koreans, women who consumed coffee more than three times a day had higher BMI and WC values than did women who consumed coffee less than once a day [31].

In one study, the type of coffee consumed was found to be associated with the odds of obesity. Instant coffee drinkers who used sugar and creamer had significantly higher risks of overweight (BMI > 23) and abdominal obesity than did non-drinkers. However, filtered coffee drinkers did not show a significant increase in overweight or abdominal obesity compared with non-drinkers [32]. In the present study, high coffee consumption with the additional intake of sugar and creamer was associated with higher obesity prevalence, as assessed by the Asian standards of BMI and WC. Therefore, differences in results among studies from various regions of the world regarding the relationship between coffee consumption and obesity might partially be due to differences in the type of coffee consumed and the use of coffee additives.

Caffeine is one of the chemicals in coffee that can affect obesity. Caffeine has been reported to induce hyperactivity of the sympathetic nervous system, thereby accelerating the consumption of energy and loss of body fat [33,34]. After 16 weeks of caffeine intake, caffeine stimulated the breakdown of fat cells and stimulated the secretion of catecholamines to increase the oxidation and metabolism of fatty acids, thereby inhibiting weight gain and body fat accumulation in animals [35]. In human studies, caffeine intake has also been found to increase heat production and energy consumption [34,36]. However, in the present study, the BMIs and WCs of Korean women increased as coffee consumption increased. This finding might be related to the fact that the most common type of coffee consumed in Korea is instant coffee mix that includes sugar and creamer [10,37]. The average amount of sugar in one serving (12 g) of instant coffee mix is 5.7 g, and the saturated fat content due to the non-dairy creamer is 1.2 g, accounting for 50% and 10% of the coffee mix by weight, respectively [38,39]. Therefore, the additional calories contained in the coffee mix might have contributed to the body weight gain of the subjects.

In this study, the risk of obesity remained after adjusting for the use of sugar and non-dairy creamer. Instant coffee and ground bean coffee manufacturing methods differ; therefore, differences in ingredient composition and content after coffee extraction may exist. However, we did not directly record the type of coffee consumed by the subjects. In previous studies, filtered coffee had different physiological effects than boiled coffee by filtering of lipophilic substances [28,40]. Further studies are warranted to identify residual confounding factors.

Women show changes in fat metabolism after menopause. Premenopausal estrogen accumulates fat in the hips and thighs, but after menopause, the estrogen deficiency redistributes body fat and promotes abdominal obesity [41]. Because coffee contains some phytoestrogen components [14], we examined the possibility that menopause modifies the effects of coffee. However, the effect of coffee consumption on abdominal obesity was not altered by menopause.

The strengths of this study are as follows. First, we performed a large-scale study to assess the relationship between coffee consumption and obesity risk in Korean women. Second, we collected data on the amount of coffee consumed and the amounts of sugar and non-dairy creamer that were added to coffee using a food frequency questionnaire. The limitations of this study are as follows. First, because this study has a cross-sectional design, the determination of a causal relationship between coffee consumption and obesity is difficult. Second, a detailed investigation of the type of coffee consumed, i.e., instant vs. brewed, was not performed at the time the FFQ was administered. Finally, because this study was conducted in participants who received a health screen, the possibility of selection bias cannot be excluded. Recently, a rapidly increasing trend toward a preference for high-quality, roasted bean brewed coffee over instant coffee mix has been noted, particularly among young people in Korea [32,37]. Given changes in coffee consumption patterns, future studies should examine potential changes in obesity prevalence rates.

## 5. Conclusions

In this cross-sectional study, frequent coffee consumption by Korean women was associated with high obesity prevalence. However, this sample of participants demonstrated additional intake of sugar and creamer with their coffee consumption, which might contribute to the increased prevalence of obesity. The positive association between coffee consumption and obesity was not altered by menopause. The importance of coffee consumption in the risk of obesity should be pursued in further studies, such as well-designed, large-scale, prospective cohort studies, in order to elucidate the causal relationship between coffee consumption and the etiology of obesity. 

## Figures and Tables

**Table 1:** General characteristics of the study participants stratified by coffee consumption category ^1^.

	Daily Coffee Consumption					
Characteristics	None (*n* = 725)	<1 Cup/Day (*n* = 1646)	1–2 Cups/Day (*n* = 1457)	2–3 Cups/Day (*n* = 1178)	≥3 Cups/Day (*n* = 989)	*p* for Trend ^2^
Age (years)	54.5 ± 8.3	53.4 ± 8.3	52.3 ± 8.2	50.2 ± 7.6	49.7 ± 7.4	<0.001
Education level						
Under middle school	168 (23.2)	310 (18.8)	202 (13.9)	115 (9.8)	100 (10.1)	<0.001
High school	282 (38.9)	642 (39.0)	584 (40.1)	491 (41.7)	406 (41.1)	
College or more	236 (32.6)	601 (36.5)	609 (41.8)	523 (44.4)	446 (45.1)	
Missing ^3^	39 (5.4)	93 (5.7)	62 (4.3)	49 (4.2)	37 (3.7)	
Occupation						
Managers and profession	70 (9.7)	161 (9.8)	147 (10.1)	173 (14.7)	138 (14.0)	<0.001
Office worker, sales, service	130 (17.9)	267 (16.2)	333 (22.9)	319 (27.1)	296 (29.9)	
Laborers, agriculture	22 (3.0)	71 (4.3)	56 (3.8)	46 (3.9)	51 (5.2)	
Not in labor force	491 (67.7)	1112 (67.6)	899 (61.7)	621 (52.7)	483 (48.8)	
Missing ^3^	12 (1.7)	35 (2.1)	22 (1.5)	19 (1.6)	21 (2.1)	
Alcohol consumption						
Non-drinker	540 (74.5)	1002 (60.9)	793 (54.4)	565 (48.0)	416 (42.1)	<0.001
Ex-drinker	39 (5.4)	97 (5.9)	73 (5.0)	58 (4.9)	61 (6.2)	
Current drinker	144 (19.9)	544 (33.1)	586 (40.2)	553 (46.9)	512 (51.8)	
Missing ^3^	2 (0.3)	3 (0.2)	5 (0.3)	2 (0.2)	0 (0.0)	
Smoking status						
Non-smoker	691 (95.3)	1551 (94.2)	1375 (94.4)	1092 (92.7)	873 (88.3)	<0.001
Ex-smoker	14 (1.9)	53 (3.2)	45 (3.1)	49 (4.2)	56 (5.7)	
Current smoker	13 (1.8)	34 (2.1)	30 (2.1)	30 (2.6)	58 (5.9)	
Missing ^3^	7 (1.0)	8 (0.5)	7 (0.5)	7 (0.6)	2 (0.2)	
Regular exercise (yes)	398 (54.9)	925 (56.2)	774 (53.1)	564 (47.9)	447 (45.2)	<0.001
Age at menarche (years)	14.9 ± 1.7	14.8 ± 1.7	14.7 ± 2.0	14.4 ± 1.7	14.5 ± 1.6	<0.001
Menopause (yes)	523 (71.1)	1094 (66.5)	890 (61.1)	598 (50.8)	486 (49.1)	<0.001
Age at menopause ^4^ (years)	49.3 ± 4.8	49.2 ± 5.0	49.4 ± 4.8	49.4 ± 4.7	49.1 ± 4.8	0.845
Postmenopausal hormone use ^4^						
Never	341 (65.2)	713 (65.2)	597 (67.1)	421 (70.4)	353 (72.6)	0.001
Ever	164 (31.4)	345 (31.5)	268 (30.1)	156 (26.1)	121 (24.9)	
Total caloric intake (kcal/day)	1562.7 ± 603.1	1576.2 ± 586.2	1629.2 ± 554.4	1666.3 ± 573.0	1763.8 ± 661.6	<0.001
Coffee intake (cups/day)	0.0 ± 0.0	0.4 ± 0.3	1.0 ± 0.1	2.0 ± 0.0	3.8 ± 1.1	<0.001
Coffee sugar addition (g/day)	0.0 ± 0.0	0.9 ± 1.5	2.1 ± 2.2	4.2 ± 3.9	5.8 ± 6.2	<0.001
Coffee creamer addition (g/day)	0.0 ± 0.0	0.5 ± 0.9	1.4 ± 1.8	3.1 ± 3.2	4.4 ± 5.0	<0.001

^1^ Data presented as unadjusted mean ± SD for continuous variables or prevalence (%) for categorical variables; ^2^ *p* for trend was calculated using the Mantel–Haenszel χ^2^ test for categorical variables and generalized linear models for continuous variables; ^3^ Missing included no response or unwilling to respond; ^4^ In postmenopausal women.

**Table 2:** Anthropometric measurements of the study participants stratified by coffee consumption category.

	Daily Coffee Consumption					
Anthropometric Factor	None (*n* = 725)	<1 Cup/Day (*n* = 1646)	1–2 Cups/Day (*n* = 1457)	2–3 Cups/Day (*n* = 1178)	≥3 Cups/Day (*n* = 989)	*p* for Trend
Height (cm)						
Model 1	156.70 ± 5.43	157.31 ± 5.25	157.42 ± 5.19	157.83 ± 5.03	158.01 ± 4.98	<0.001
Model 2	157.31 ± 5.09	157.65 ± 4.89	157.42 ± 4.89	157.51 ± 4.82	157.59 ± 4.64	0.583
Model 3	157.15 ± 5.09	157.54 ± 4.89	157.40 ± 4.89	157.62 ± 4.82	157.79 ± 4.61	0.186
Weight (kg)						
Model 1	55.18 ± 7.22	57.47 ± 7.64	57.61 ± 7.61	58.32 ± 7.72	58.62 ± 7.69	<0.001
Model 2	55.12 ± 7.24	57.57 ± 7.73	57.51 ± 7.50	58.36 ± 7.59	58.53 ± 7.58	<0.001
Model 3	54.80 ± 7.24	57.35 ± 7.73	57.45 ± 7.48	58.57 ± 7.59	58.93 ± 7.54	<0.001
Body mass index (kg/m^2^)						
Model 1	22.49 ± 2.88	23.23 ± 2.94	23.27 ± 3.08	23.42 ± 3.00	23.49 ± 2.99	<0.001
Model 2	22.29 ± 2.82	23.17 ± 2.94	23.23 ± 2.96	23.53 ± 2.84	23.57 ± 2.85	<0.001
Model 3	22.20 ± 2.82	23.12 ± 2.94	23.21 ± 2.96	23.58 ± 2.84	23.68 ± 2.84	<0.001
Waist circumference (cm)						
Model 1	72.97 ± 7.42	74.36 ± 7.33	74.35 ± 7.44	74.29 ± 7.53	74.36 ± 7.65	<0.001
Model 2	72.25 ± 6.87	74.09 ± 7.11	74.23 ± 6.87	74.66 ± 7.03	74.71 ± 2.28	<0.001
Model 3	72.11 ± 6.87	73.99 ± 7.11	74.20 ± 6.86	74.76 ± 7.04	74.91 ± 7.26	<0.001

Data presented as adjusted mean ± SD. Model 1 was unadjusted. Model 2 was adjusted for age, education level, occupation, alcohol intake, smoking status, regular exercise, total energy intake. Model 3 was adjusted for covariates in Model 2 + sugar and creamer additive use.

**Table 3:** Odds ratio (OR) and 95% confidence interval (CI) for the prevalence of obesity as defined by body mass index (≥25) according to coffee consumption category.

Daily Coffee Consumption	No of Subjects		Model 1	Model 2	Model 3
	Without Obesity ^1^	With Obesity			
All subjects					
None	612 (13.4)	113 (7.9)	1.00 (ref)	1.00 (ref)	1.00 (ref)
<1 cup/day	1269 (27.8)	377 (26.4)	1.61 (1.28–2.03)	1.76 (1.39–2.23)	1.75 (1.38–2.22)
1–2 cups/day	1096 (24.0)	361 (25.3)	1.78 (1.41–2.25)	2.05 (1.61–2.60)	2.04 (1.60–2.60)
2–3 cups/day	874 (19.1)	304 (21.3)	1.88 (1.48–2.39)	2.36 (1.84–3.03)	2.35 (1.81–3.05)
≥3 cups/day	717 (15.7)	272 (19.1)	2.06 (1.61–2.62)	2.54 (1.96–3.28)	2.52 (1.91–3.34)
*p* for trend ^2^			<0.001	<0.001	<0.001
Premenopausal women					
None	177 (9.2)	25 (5.3)	1.00 (ref)	1.00 (ref)	1.00 (ref)
<1 cup/day	452 (23.4)	100 (21.1)	1.57 (0.98–2.51)	1.61 (0.99–2.60)	1.63 (1.00–2.64)
1–2 cups/day	458 (23.7)	109 (23.0)	1.69 (1.06–2.69)	1.70 (1.05–2.75)	1.75 (1.08–2.85)
2–3 cups/day	458 (23.7)	122 (25.7)	1.89 (1.19–3.00)	1.89 (1.17–3.06)	2.00 (1.22–3.27)
≥3 cups/day	385 (20.0)	118 (24.9)	2.17 (1.36–3.46)	2.10 (1.29–3.41)	2.28 (1.36–3.82)
*p* for trend ^2^			0.001	0.007	0.006
Postmenopausal women					
None	435 (16.5)	88 (9.2)	1.00 (ref)	1.00 (ref)	1.00 (ref)
<1 cup/day	817 (31.0)	277 (29.1)	1.68 (1.28–2.19)	1.79 (1.36–2.35)	1.76 (1.34–2.32)
1–2 cups/day	638 (24.2)	252 (26.4)	1.95 (1.49–2.56)	2.16 (1.63–2.86)	2.10 (1.58–2.79)
2–3 cups/day	416 (15.8)	182 (19.1)	2.16 (1.62–2.88)	2.52 (1.87–3.39)	2.40 (1.75–3.30)
≥3 cups/day	332 (12.6)	154 (16.2)	2.29 (1.70–3.09)	2.66 (1.95–3.64)	2.52 (1.79–3.54)
*p* for trend ^2^			<0.001	<0.001	<0.001

Model 1 was unadjusted. Model 2 was adjusted for age, education level, occupation, alcohol intake, smoking status, regular exercise, total energy intake. Model 3 was adjusted for covariates in Model 2 + sugar and creamer additive use. ^1^ Obesity was defined as a body mass index of ≥25 kg/m^2^; ^2^ *p* for trend was calculated using the median value of each category as a continuous variable.

**Table 4:** Odds ratio (OR) and 95% confidence interval (CI) for the prevalence of abdominal obesity as defined by waist circumference according to coffee consumption category.

Daily Coffee Consumption	No of Subjects		Model 1	Model 2	Model 3
	Without Obesity ^1^	With Obesity			
All subjects					
None	597 (12.8)	128 (9.6)	1.00 (ref)	1.00 (ref)	1.00 (ref)
<1 cup/day	1275 (27.3)	371 (27.9)	1.36 (1.09–1.70)	1.52 (1.21–1.92)	1.52 (1.21–1.92)
1–2 cups/day	1122 (24.1)	335 (25.2)	1.39 (1.11–1.75)	1.70 (1.34–2.15)	1.71 (1.34–2.17)
2–3 cups/day	916 (19.6)	262 (19.7)	1.33 (1.05–1.69)	1.88 (1.47–2.41)	1.93 (1.48–2.51)
≥3 cups/day	755 (16.2)	234 (17.6)	1.45 (1.14–1.84)	2.03 (1.57–2.63)	2.11 (1.59–2.79)
*p* for trend ^2^			0.083	<0.001	<0.001
Premenopausal women					
None	185 (9.1)	17 (4.5)	1.00 (ref)	1.00 (ref)	1.00 (ref)
<1 cup/day	466 (23.0)	86 (22.9)	2.01 (1.16–3.47)	2.16 (1.23–3.78)	2.18 (1.25–3.83)
1–2 cups/day	485 (23.9)	82 (21.9)	1.84 (1.06–3.19)	1.96 (1.11–3.44)	2.00 (1.14–3.54)
2–3 cups/day	484 (23.9)	96 (25.6)	2.16 (1.25–3.71)	2.36 (1.35–4.14)	2.51 (1.41–4.46)
≥3 cups/day	409 (20.2)	94 (25.1)	2.50 (1.45–4.31)	2.59 (1.47–4.55)	2.82 (1.55–5.12)
*p* for trend ^2^			0.007	0.012	0.010
Postmenopausal women					
None	412 (15.6)	111 (11.6)	1.00 (ref)	1.00 (ref)	1.00 (ref)
<1 cup/day	809 (30.7)	285 (29.8)	1.31 (1.02–1.68)	1.40 (1.08–1.82)	1.40 (1.08–1.81)
1–2 cups/day	637 (24.2)	253 (26.5)	1.47 (1.14–1.90)	1.66 (1.27–2.17)	1.67 (1.27–2.19)
2–3 cups/day	432 (16.4)	166 (17.4)	1.43 (1.08–1.88)	1.76 (1.32–2.35)	1.79 (1.31–2.44)
≥3 cups/day	346 (13.1)	140 (14.7)	1.50 (1.13–2.00)	1.87 (1.37–2.54)	1.90 (1.36–2.67)
*p* for trend ^2^			0.023	<0.001	0.001

Model 1 was unadjusted. Model 2 was adjusted for age, education level, occupation, alcohol intake, smoking status, regular exercise, total energy intake. Model 3 was adjusted for covariates in Model 2 + sugar and creamer additive use. ^1^ Obesity was defined as a waist circumference of ≥80 cm; ^2^ *p* for trend was calculated using the median value of each category as a continuous variable.
